# Preoperative administration of branched-chain amino acids reduces postoperative insulin resistance in rats by reducing liver gluconeogenesis

**DOI:** 10.1186/s12986-022-00710-3

**Published:** 2022-11-29

**Authors:** Jin Zhang, Rui Chi, Yunpeng Zhang, Yi Xie, Yunxia Liu, Qun Bao, Hengyu Lv, Bo Han, Haipeng Sun, Peng Sun

**Affiliations:** 1 Department of General Surgery, Tongren Hospital, Shanghai Jiao Tong University School of Medicine, Shanghai, 200336 China; 2 Department of Pathophysiology, Key Laboratory of Cell Differentiation and Apoptosis of Chinese Ministry of Education, Shanghai Jiao Tong University School of Medicine, Shanghai, 200025 China; 3 Hongqiao International Institute of Medicine, Tongren Hospital, Shanghai Jiao Tong University School of Medicine, Shanghai, 200336 China; 4 NHC Key Laboratory of Hormones and Development, Center for Cardiovascular Diseases, The Province and Ministry Cosponsored Collaborative Innovation Center for Medical Epigenetics, Chu Hsien-I Memorial Hospital and Tianjin Institute of Endocrinology, Tianjin Medical University, Tianjin, 300134 China; 5 Department of Pathophysiology, Key Laboratory of Cell Differentiation and Apoptosis of Chinese Ministry of Education, Hongqiao International Institute of Medicine, Tongren Hospital, Shanghai Jiao Tong University School of Medicine, Shanghai, 200025 China

**Keywords:** Branched-chain amino acids, Postoperative insulin resistance, Gluconeogenesis, CRTC2, G6PC, Hyperglycemia

## Abstract

**Background:**

Postoperative insulin resistance (PIR) represents an important characteristic of metabolic response following surgical injury. Clinical outcomes are negatively correlated to postoperative insulin resistance and hyperglycemia, indicating a novel treatment for reducing postoperative insulin resistance is urgently needed. The current work aimed to assess the protective effects of branched-chain amino acids (BCAA) on glucose metabolism disorders induced surgically in a rat model, and to explore the underpinning mechanism.

**Methods and results:**

Rats were randomly assigned to 2 groups, including the control and BCAA groups. Rats were given a compulsory oral 3 mL load by gavage two hours before surgery. The results showed that BCAA remarkably reduced glycemia by suppressing liver gluconeogenesis via reduction of cAMP-response element-binding protein-regulated transcription coactivator 2 (CRTC2) and glucose-6-phosphatase (G6PC) gene and protein expression levels (all *Ps* < 0.05).

**Conclusions:**

This study revealed that BCAA lower blood glucose levels by reducing liver gluconeogenesis without significant elevation of plasma insulin levels. We anticipate that preoperative BCAA supplementation may be a means for preventing postoperative insulin resistance.

**Supplementary Information:**

The online version contains supplementary material available at 10.1186/s12986-022-00710-3.

## Background

Postoperative insulin resistance (PIR) represents an important characteristic of metabolic response following surgical injury [1]. The main manifestations of PIR include elevated blood glucose, abnormal glucose tolerance, inadequate body response to conventional doses of insulin therapy, and development of uncontrollable hyperglycemia. PIR is present in almost all kinds of surgery and occurs even after a minor elective surgery such as inguinal hernia repair. The degree of PIR is related to the size of the surgical trauma, for example, the mean relative reduction in insulin sensitivity after laparoscopic surgery was significantly smaller compared to open surgery. Elective surgery (a kind of surgery that can be performed at an appropriate time, allowing for adequate preparation or observation prior to surgery so that the most favorable time can be chosen for the procedure) causes transient insulin resistance, which is most prominent the day following surgery and could last for about 2 to 3 weeks [2]. PIR can increase the incidence of postoperative complications and patient mortality [3, 4]. Reports have demonstrated PIR is an independent risk factor for patient prognosis that leads to prolonged hospital stay and increased costs [5, 6]. Clinical outcomes are negatively correlated with postoperative insulin resistance and hyperglycemia [4, 5, 7]. Multiple studies reported an association of postoperative hyperglycemia with elevated risk of surgery-related infections [4]. Therefore, strict blood glucose monitoring is crucial as well as prompt hyperglycemia treatment. Based on the current understanding of the mechanism of postoperative insulin resistance, useful clinical attempts have been made, and measures that have been utilized to prevent and treat postoperative insulin resistance in recent years mainly include preoperative oral carbohydrates, epidural anesthesia, effective postoperative analgesia and minimally invasive surgical techniques [8–10]. Although these measures alleviate PIR to some extent clinically, they cannot completely prevent its occurrence. At present, the etiological mechanism of acute insulin resistance (AIR) is unclear, and much less research has been conducted on the development of AIR compared with insulin resistance associated with chronic diseases. Therefore, it is important to assess the mechanisms of PIR and develop new treatment methods to clinically address PIR occurrence, reduce postoperative hyperglycemia and decrease the incidence of postoperative complications.

Branched-chain amino acids (BCAA), including valine, leucine and isoleucine, constitute essential nutrients for humans [11]. Recent studies have accumulated evidence that BCAA have major regulatory functions in protein metabolism and glucose homeostasis, and growing evidence suggests a strong link between BCAA and blood glucose levels [12–17]. Interestingly, studies have shown BCAA supplementation and BCAA-rich foods positively modulate body weight and glucose homeostasis [18–21]. A study showed that BCAA supplementation restores reduced insulin activity in rats on a low-protein diet via the vagus nerve [22]. In clinical chronic viral liver disease, BCAA reduce insulin resistance [23]. Recent studies have shown that leucine improves glucose uptake in the muscle tissue [24], and increasing dietary leucine content significantly improves glucose tolerance and insulin signaling [25]. It has been found in rodent assays that isoleucine, an isomer of leucine, is more effective than leucine in suppressing blood glucose concentration increase [26–29]. The above data indicate BCAA might contribute to the regulation of glucose metabolism. Notably, clinical studies in recent years have found that disorders of BCAA metabolism are closely related to the development of diabetes, and serum BCAA levels could even predict the development of diabetes and prognosis after treatment [30, 31]. Basic studies, on the other hand, have reported that high BCAA amounts have two distinct effects of promoting and inhibiting glucose metabolism, for reasons that remain unclear, which is currently one of the hot topics in the related field. Preoperative oral carbohydrate (POC) is a measure adopted in Enhanced Recovery After Surgery (ERAS) in recent years, which has been shown to reduce PIR [32–35]. In the last decade or so, many national anesthesia societies have revised their anesthesia guidelines to shorten the duration of preoperative fasting and to promote the consumption of 400 mL of 12.5% carbohydrates 2 hours before surgery. Related studies have demonstrated that POC can shorten preoperative fasting time, reduce PIR, decrease resting energy expenditure, improve substance metabolism, and accelerate patient recovery. POC is recommended by several ERAS guidelines, including the ASA Committee, ERAS Society, and ESPEN. Based on the latter findings, we wondered whether preoperative oral BCAA could also affect PIR.

The liver is the major organ that produces glucose via gluconeogenesis and glycogenolysis [36]. Reduced gluconeogenesis in the liver is a possible mechanism by which amino acids lower blood glucose levels. In the fasting state, glucose production mainly results from gluconeogenesis. cAMP-response element-binding protein (CREB)-regulated transcription coactivator 2 (CRTC2), a coactivator with specificity to CREB, plays important roles in regulating gluconeogenesis in the liver and maintaining the stability of fasting blood glucose [37]. Dephosphorylation of CRTC2 Ser171 results in its activation and binding to CREB, which in turn binds to glucose-6-phosphatase (G6PC) and phosphoenolpyruvate carboxykinase 1 (PCK1), key downstream enzymes regulating the rate of gluconeogenesis, to promote hepatic gluconeogenesis [38, 39]. IL-6, a major cytokine that promotes inflammation, has been shown in many studies to have a close relationship with the development of postoperative insulin resistance [40, 41]. A recent study showed IL-6 is also a key factor in promoting gluconeogenesis, and endocrine IL-6 is a directive signal necessary to mediate hyperglycemia through hepatic gluconeogenesis during stress [42].

Currently it is not known whether BCAA modulate glucose metabolism. In this study, we used a rat model of small bowel resection. Similar to preoperative oral carbohydrate, preoperative administration of BCAA was carried out to examine the effect on PIR. This work aimed to assess the contribution of preoperatively administered BCAA on gluconeogenesis and CREB/CRTC2 signaling in PIR reduction.

## Materials and methods

### Chemicals

BCAA were provided by Sigma (USA) and dissolved in double distilled water. D-glucose (Sigma), insulin (Beyotime Biotechnology, China) and pyruvic acid (Sangon Biotech, China) were utilized in this study.

### Animals

Six-week-old male Sprague–Dawley (SD) rats (180–200 g) were provided by Charles River (China) and housed at ambient temperature (22℃) under a 12 h/12 h light–dark cycle with food and water ad libitum. After adaptive housing, the animals underwent a 17-h fasting prior to surgery starting at 5:00 pm. The animals were randomized into 2 groups of 8, including the control (CON) and BCAA groups, orally administered double distilled water and BCAA (0.375 g/kg body weight of leucine: isoleucine: valine at 1:1:1), respectively. The rats were administered a compulsory oral 3 mL load by gavage two hours before surgery. Euthanasia was carried out by exposure to CO2, followed by decapitation. The present study had approval from the Laboratory Animal Welfare Ethics branch of the Biomedical Ethics Committee of Tongren Hospital, Shanghai Jiaotong University School of Medicine (Approval No. A2022-007-01).

### PIR surgery model in rats

**Fig. 1:**
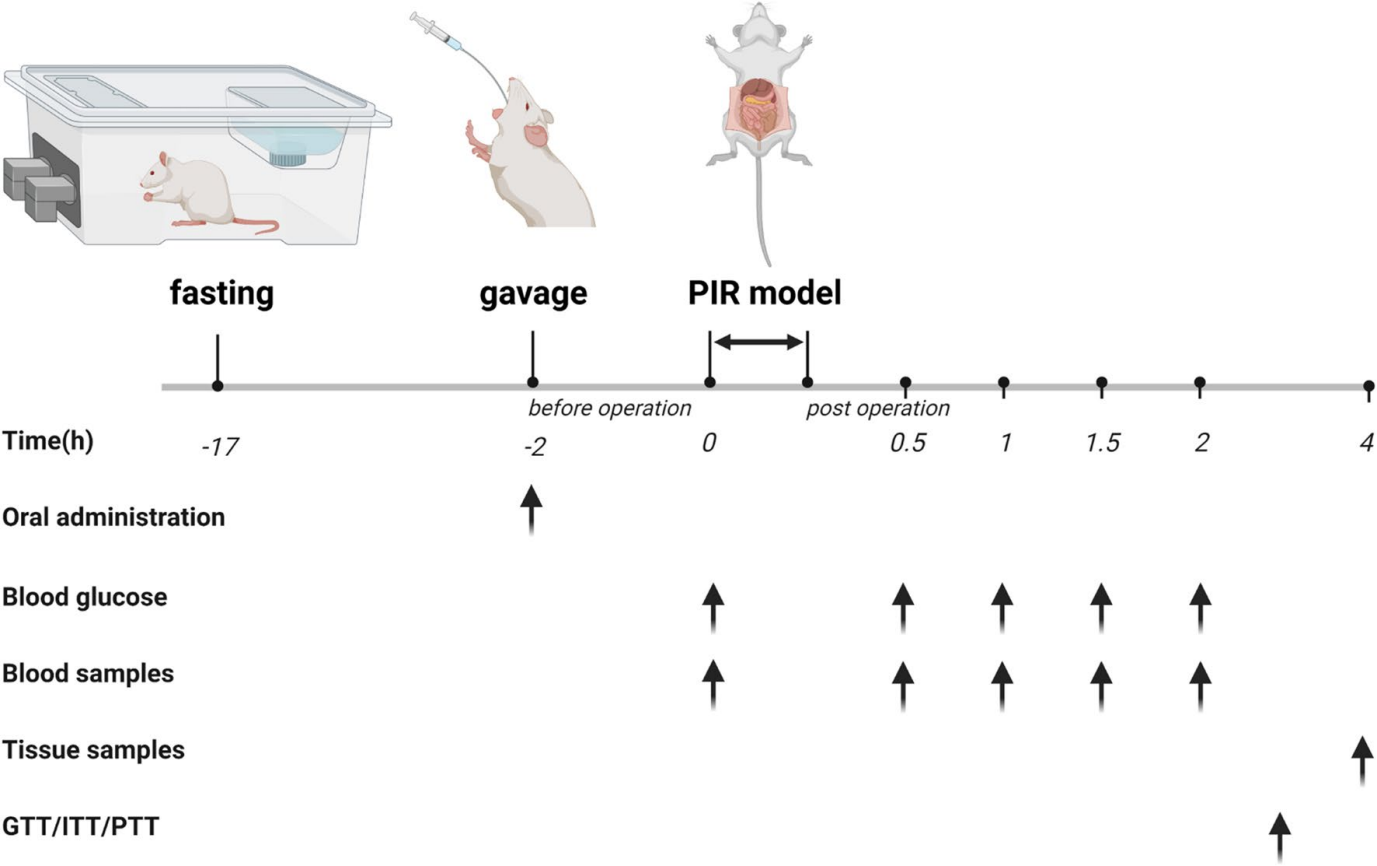
The schematic diagram of the experiment protocol. Rats were fasted for 17 h be-fore surgery and received BCAA or CON oral administration by gavage 2 h prior to surgery. Rats were anesthetized with isoflurane gas and then treated with the PIR model. Blood glucose was measured immediately according to the time points in the diagram. Blood samples and tis-sue samples were collected as shown in the diagram, and for further testing. GTT/ITT/PTT were measured using another group of rats. PIR: Postoperative insulin resistance, BCAA: branched-chain amino acids, CON: control, GTT: glucose tolerance test, ITT: insulin tolerance test, PTT: pyruvate tolerance test

Rats were anesthetized with isoflurane 2 h after BCAA or CON gavage. After fixation in the dorsal position, the rat abdomen was shaved and disinfected. Then, the right carotid artery was cannulated to collect blood. After a 5-cm midline abdominal incision to expose the small bowel, 2–3 mesenteric vessels were separated 5 cm from the Treitz ligament. The 5-cm long small bowel underwent excision. Small bowel anastomosis with 6/0 sutures in the abdomen [43]. The surgery lasted 30 min on average. Post-surgically, the animals were maintained in individual cages under a heat lamp. Blood specimens (300 μL) were obtained preoperatively, and 30, 60, 90 and 120 min post-surgically, for immediate blood glucose measurements. Then, blood specimens underwent a 20-min centrifugation (4 °C at 3000 rpm) for serum collection; serum specimens were kept at − 80 °C for further measurements. Euthanasia was carried out 4 h postoperatively. Then, epididymal fat, skeletal muscle and liver tissue specimens were immediately collected, snap frozen in liquid nitrogen and stored at − 80 °C until utilization (Fig. 1).

### Measurement of blood insulin and glucagon

Plasma samples were kept at − 80 °C until utilization. Insulin and glucagon levels were assessed with an Ultrasensitive Rat Insulin ELISA Kit (Beyotime Biotechnology; PI606) and a Rat Glucagon ELISA Kit (Beyotime Biotechnology; PG357), respectively, as directed by the manufacturer.

### Insulin resistance assessment

We used the homeostasis model assessment (HOMA) model [44] for assessing insulin resistance-related indexes. Insulin resistance was determined with the HOMA of insulin resistance (HOMA-IR) as blood glucose (mM) × plasma insulin (μ IU/mL)/22.5. Insulin secretion index was obtained as HOMA-β by using the following formula: 20 × plasma insulin (μ IU/mL)/ [blood glucose (mM)—3.5]. Insulin sensitivity index was obtained as HOMA-ISI using the following formula: ln [22.5/ blood glucose (mM) × plasma insulin (μ IU/mL)].

### Glucose, insulin, and pyruvate tolerance tests

After two hours in the PIR model, rats were intraperitoneally injected D-glucose (2 g/kg), insulin (1 U/kg) and sodium pyruvate (2 g/kg) for the glucose-tolerance (GTT), insulin-tolerance (ITT) and pyruvate tolerance (PTT) tests, respectively. These rats were not used for tissue extraction (n = 8 per group). Blood glucose content was assessed with a glucometer (Johnson & Johnson, USA) before the injection and at 30, 60, 90 and 120 min thereafter. Each glucose curve was analyzed, and the total area under the curve (AUC) was determined with GraphPad Prism.

### Multiplex cytokine assay

Serum specimens were examined with the rat multiplex cytokine kit (LXSARM-08; R&D Systems, USA), which allows simultaneous assessment of interleukin (IL)-1α, IL-1β, IL-4, IL-6, IL-10, tumor necrosis factor-α (TNF-α), interferon γ (IFN-γ) and Granulocyte–Macrophage Colony-Stimulating Factor (GM-CSF). The assay was carried out as directed by the manufacturer. Finally, the plate was read on a Luminex 200 (Luminex, USA) plate reader.

### RNA isolation and quantitative RT-PCR

Total mRNA extraction from various tissue specimens utilized TRIzol reagent (15596018, Invitrogen, USA), as directed by the manufacturer. First strand cDNA was obtained with a revere transcription kit (FSO-301, TOYOBO, Japan) and amplified with SYBR Premix Ex Taq (RR420A, Takara, Tokyo, Japan). The signal was detected with the QuantStudio Design & Analysis Software. The utilized primers are described in Table 1. The 2^−∆∆Ct^ method was used for data analysis.

**Table 1:** Details of qPCR primers

Gene abbreviation	Forward sequence	Reverse sequence
GAPDH	GGGTGTGAACCACGAGAAAT	ACTGTGGTCATGAGCCCTTC
SREBP-1c	GGAGCCATGGATTGCACATT	AGGCCAGGGAAGTCACTGTCT
FBP1	CCATCATAATAGAGCCCGAGAAGA	CTTTCTCCGAAGCCTCATTAGC
PI3K	ATGCAACTGCCTTGCACATT	ACTGGCAATCTCAGCTGCC
AKT	GCCCAACACCTTCATCATCC	GTCTCCTCCTCCTGCCGTTT
FoxO1	TCCTCGAACCAGCTCAAACG	GGCGGTGCAAATGAATAGCAAG
CRTC2	AGGCCTGCTTAGTACAGCCCT	GGCCACTCACTGATCCCTCA
CBP	TAATGGAGGCTGCCCAGTGTGTAA	CTGGCGGAGCTTGTGTTTGATGTT
mTOR	GTGTGGCAAGAGCGGCAGAC	TGTTGGCAGAGGATGGTCAAGTTG
P70S6K	CAGACTCCACCAATCCACAGCAC	TTGCGTGTGCGAAGGAAGAACC
AMPKα1	TTGCGTGTGCGAAGGAAGAACC	CCGATCTCTGTGGAGTAGCAGTCC
AMPKα2	ATGATGAGGTGGTGGAGCAGAGG	GTTCTCGGCTGTGCTGGAATCG
FAS	ATGGCTGTCCTGCCTCTGGT	ACGCTCCTCTTCAACTCCAAA
ACC	GAGATTTCACTGTGGCTTCGC	TAATTGTTGTTGTTTGCTCCTCC
SIRT1	TGACCTCCTCATTGTTATTGG	GGCATACTCGCCACCTAA
CREB	CTGATTCCCAAAAACGAAGG	CTGCCCACTGCTAGTTTGGT
PCK1	CAGGATCGAAAGCAAGACGGT	AAGTCCTCTTCTGACATCCAG
G6PC	GACTGGTTCAACCTCGTCTTT	GTCTCACAGGTGACGGGGAAC
PPAR-a	ACAAGGCCTCAGGATACCA	GCCGAAAGAAGCCCTTGCAG
GLUT2	AGACAACAACTCCGCACG	TCCAGAGGAACACCCAAAAC
GCK	GTGTACAAGCTGCACCCGA	CAGCATGCAAGCCTTCTTG
Chrebp	ACAAGAAGCGGCTCCGTAAGTCC	GGGGGCGATAATTGGTGAAGAAA

### Protein extraction and western blot analysis

Approximately 50 mg of tissue samples were mixed with the RIPA lysis buffer, homogenized and centrifuged at 4 °C and 12,000 rpm (20 min). The Pierce BCA Protein Assay kit (Thermo Scientific, USA) was used to quantitate protein amounts in the resulting supernatants. Equal amounts of total protein (30 µg) underwent separation by sodium dodecyl sulfate–polyacrylamide gel electrophoresis (SDS-PAGE), transfer onto PVDF membranes, and successive incubations with primary and secondary antibodies. An enhanced chemiluminescence kit (Pierce, USA) was utilized for development. ImageJ and GraphPad Prism 7.0 were employed for quantitation. Primary antibodies are listed in Table 2.

**Table 2:** Details of primary antibody used in the experiment

Antibodies	Source	Catalogue no	Dilution ratio
PGC1α	Abcam	ab191838	1:1000
CRTC2	Proteintech	12497-1-AP	1:500
p-CRTC2 (Ser171)	Abcam	ab203187	1:500
SIRT1	CST	9475S	1:1000
GLUT2	Abcam	ab192599	1:1000
p-FoxO1 (Ser256)	CST	9461T	1:1000
FoxO1	CST	2880T	1:1000
CBP	CST	7389S	1:1000
G6PC	Abcam	ab133964	1:1000
FBP1	Abcam	ab109732	1:1000
p-AMPK(α1 + α2)	Abcam	ab133448	1:1000
p-PI3K(Y607)	Abcam	ab182651	1:1000
PCK1	Abcam	ab70358	1:1000
CREB	CST	9197S	1:1000
p-CREB (Ser133)	CST	9198S	1:1000
GCKR	CST	14328S	1:1000
p-AKT(Ser473)	CST	4051S	1:1000
p-AKT(Thr308)	CST	2965S	1:1000
AKT	CST	9272S	1:1000
p-P70S6K(Thr389)	CST	9206s	1:1000
β-actin-HRP	Abways	AB2001	1:5000

### Statistical analysis

Data were presented as mean ± standard error of the mean (SEM). Statistical comparisons were carried out by unpaired Student’s t test or two-way ANOVA with post hoc Sidak’s test. GraphPad Prism 7.0 for Windows was used for data analysis. *P* < 0.05 indicated statistical significance.

## Results

### BCAA administration reduces postoperative hyperglycemia and improves postoperative insulin resistance

**Fig. 2:**
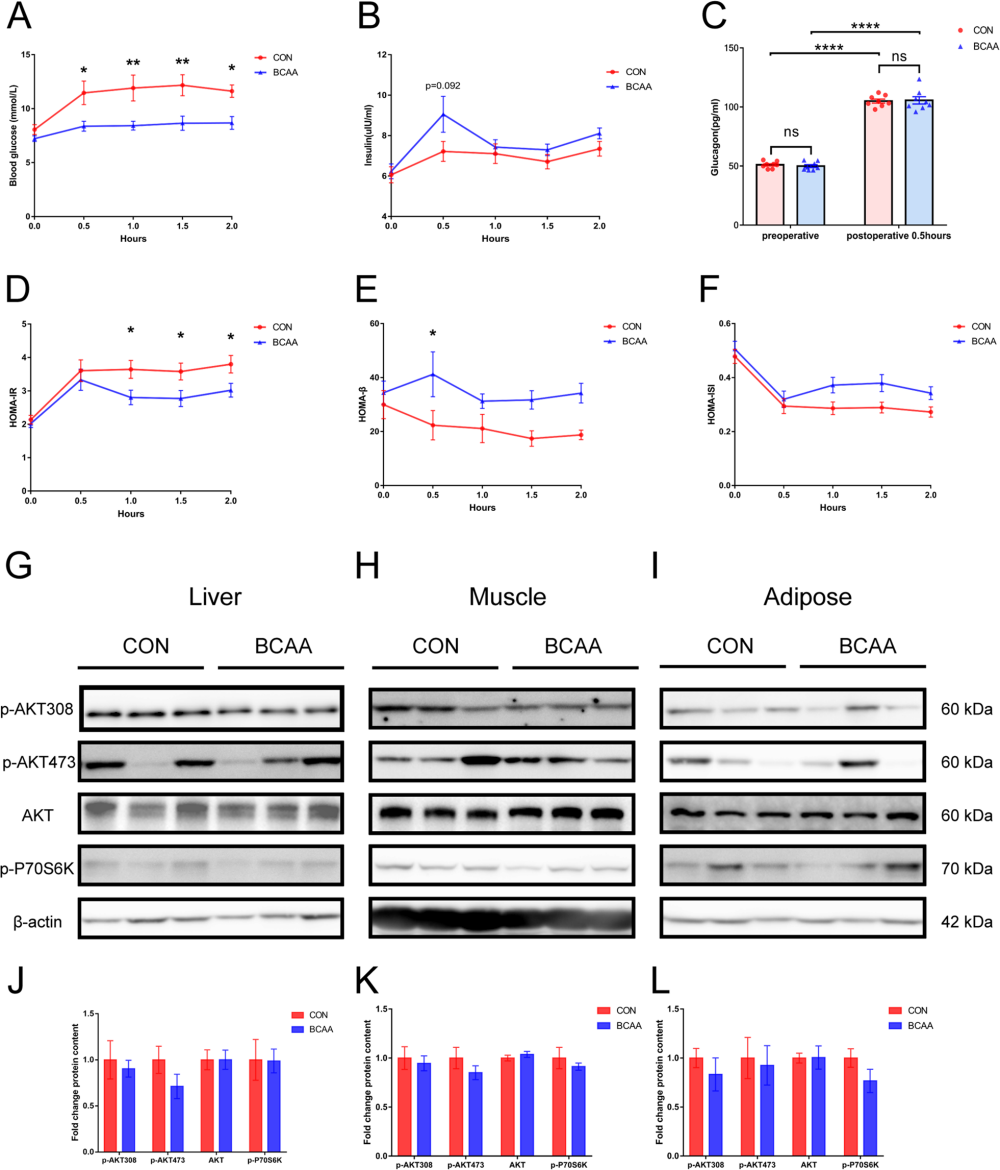
BCAA administration reduced postoperative hyperglycemia and improved postoperative insulin resistance. **A** Blood glucose, **B** plasma insulin and **C** plasma glucagon are shown(n = 8). **D** HOMA‐IR, **E** HOMA-β and** F** HOMA-ISI indices were calculated from blood glucose and plasma insulin level (n = 8). The protein levels of p-AKT308, p-AKT473, AKT, p-P70S6K in **G** liver, **H** muscle and **I** adipose tissue were analyzed by Western Blot and quantified using ImageJ software (**J, K, L**), β-actin was used as an endogenous control (n = 6). Data are shown as mean ± SEM. *P < 0.05, **P < 0.01, ****P < 0.0001, ns, not significant. BCAA: branched-chain amino acids, CON: control, AKT: Protein kinase B, P70S6K: P70 ribosomal protein S6 kinase

BCAA administered by gavage 2 h before surgery significantly reduced postoperative blood glucose(*P* < 0.05; Fig. 2A). We also did sham surgical groups, including those treated with BCAA or not. We measured blood glucose before and two hours after surgery in four groups of rats, including the sham-operated group, the sham-operated BCAA-treated group, the operated group, and the operated BCAA-treated group (n = 8). The results are added to the Additional file 1. Compared with the CON group, insulin levels tended to increase at 0.5 h postoperatively in the BCAA group (Fig. 2B), but there was no statistical significance; in addition, insulin levels were similar in both groups at the remaining time points. Meanwhile, in comparison to control animals, glucagon levels at 0.5 h postoperatively were not significantly different in the BCAA group (Fig. 2C), although both groups had significantly higher values than detected before surgery (*P* < 0.0001). BCAA administration by gavage decreased HOMA-IR at 1, 1.5 and 2 h postoperatively (*P* < 0.05, Fig. 2D) and increased HOMA-β at 0.5 h postoperatively (*P* < 0.05, Fig. 2E). However, BCAA did not affect postoperative insulin signaling pathways in the liver, muscle and adipose tissue postoperatively (Fig. 2G–L), suggesting that BCAA administration did not lower postoperative blood glucose through insulin signaling pathways.

### BCAA affects glucose, insulin, and pyruvate tolerance tests results in the PIR model

**Fig. 3:**
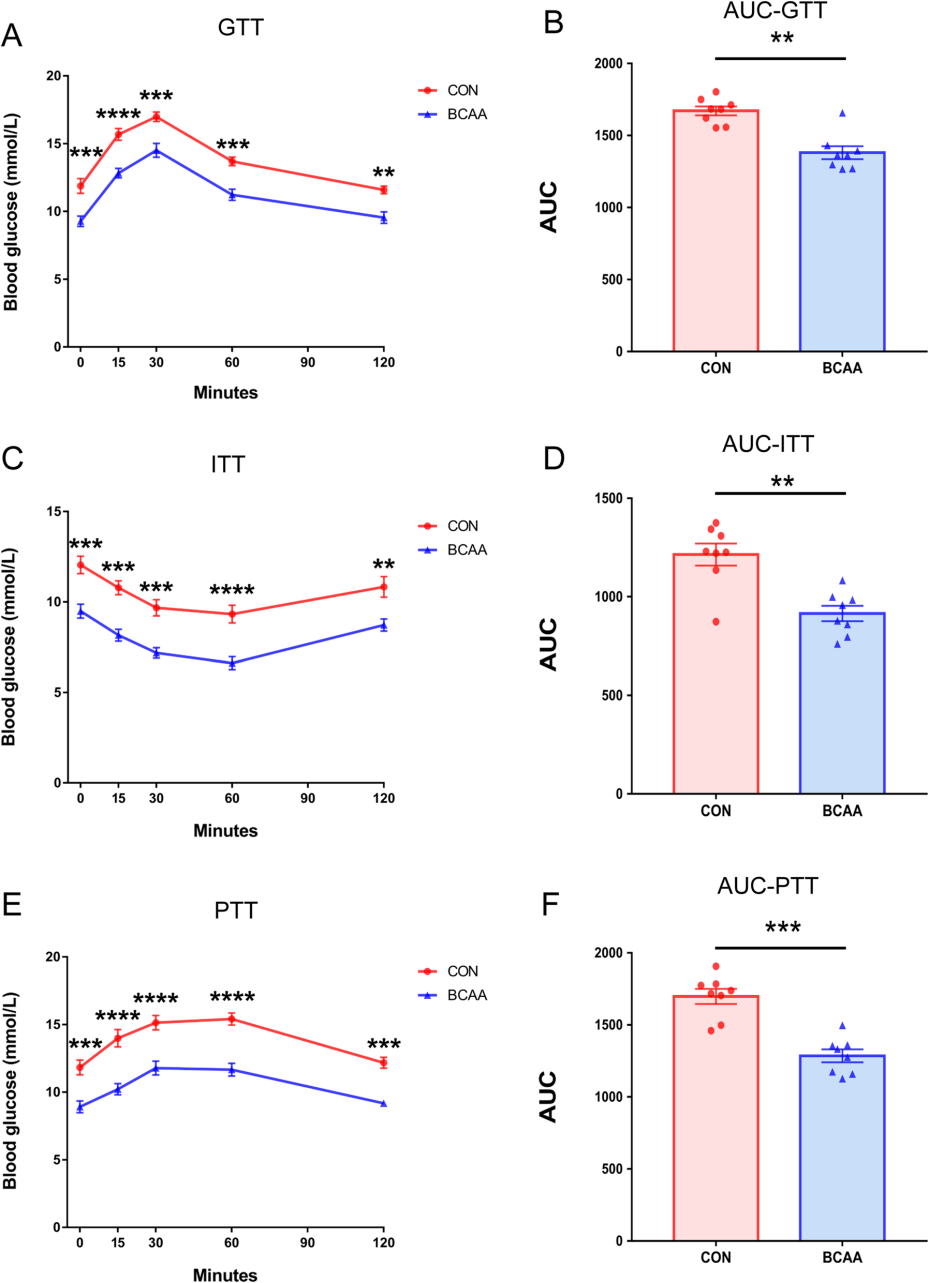
BCAA affects glucose, insulin, and pyruvate tolerance tests results in the PIR model. Two hours after the PIR model, **A** GTT, **C** ITT and **E** PTT were performed (n = 8). **B** GTT area under curve (AUC), **D** ITT AUC and **F** PTT AUC were calculated using GraphPad Prism 7.0 (n = 8). Data are shown as mean ± SEM. **P < 0.01, ***P < 0.001, ****P < 0.0001. BCAA: branched-chain amino acids, CON: control

To assess the differences in glucose metabolism between the CON and BCAA groups, glucose tolerance test (GTT), insulin tolerance test (ITT) and pyruvate tolerance test (PTT) were performed. The GTT demonstrated glucose tolerance was markedly elevated in the BCAA group in comparison with control animals (Fig. 3A), with a significant difference in AUC-GTT (Fig. 3B). The ITT revealed insulin sensitivity was starkly increased in BCAA treated animals compared with controls (Fig. 3C), with AUC-ITT showing a significant difference (Fig. 3D). In the PTT, gluconeogenesis was markedly reduced in BCAA treated animals in comparison with the control group (Fig. 3E), with a significant difference in AUC-PTT (Fig. 3F), suggesting that gavage with BCAA reduced postoperative gluconeogenesis.

***Effects of BCAA administration on cytokine levels in the PIR model.***

**Fig. 4:**
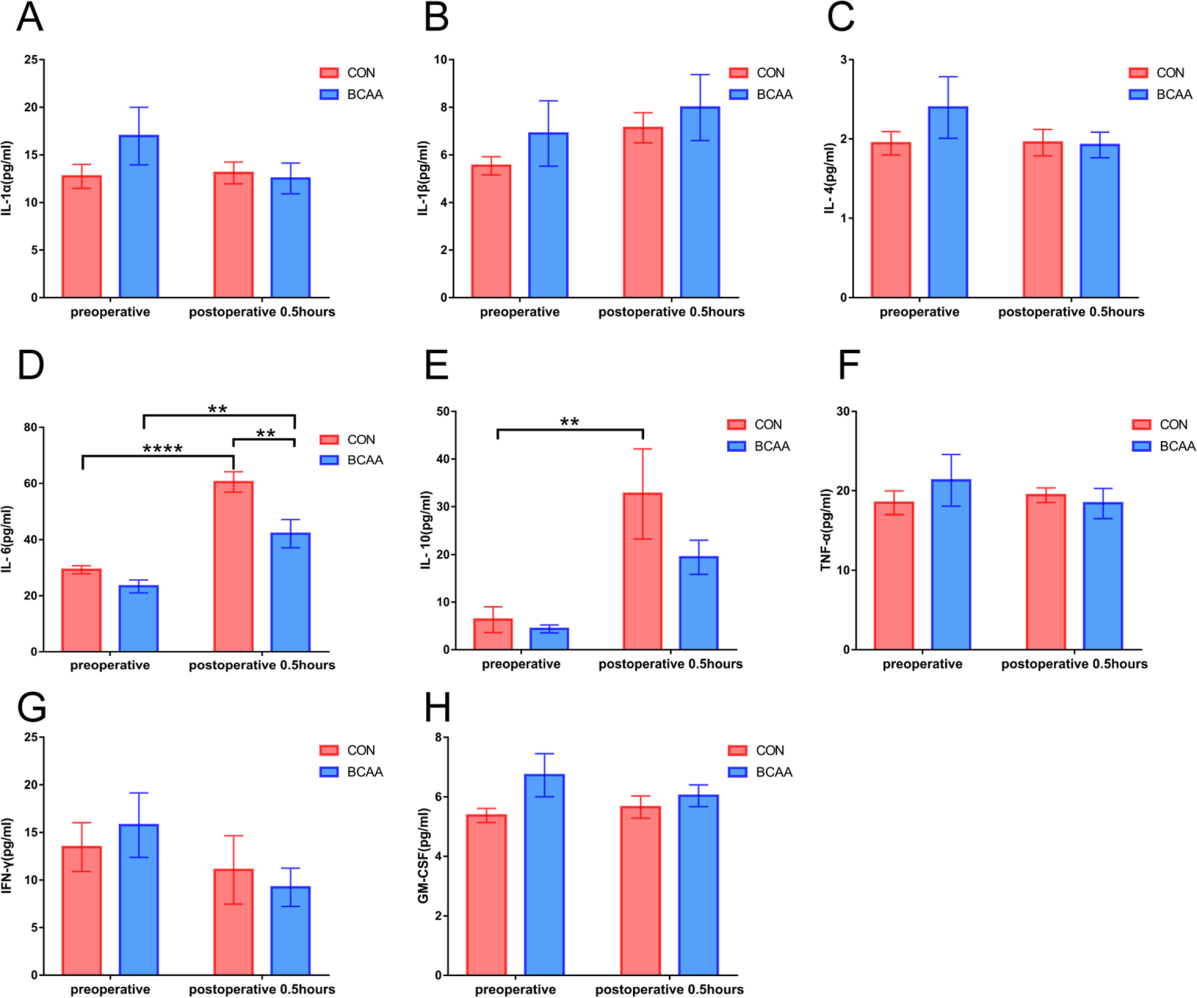
Effects of BCAA administration on cytokine levels in the PIR model. **A** IL-1α (inter-leukin-1α), **B** IL-1β (interleukin-1β), **C** IL-4(interleukin-4), **D** IL-6(interleukin-6), **E** IL-10(interleukin-10), **F** TNF-α (tumor necrosis factor-α), **G** IFN-γ (interferon γ) and **H** GM-CSF (Granulocyte–Macrophage Colony-Stimulating Factor) were determined using the Luminex assay (n = 8). Data are shown as mean ± SEM. **P < 0.01, ****P < 0.0001. BCAA: branched-chain amino acids, CON: control

Inflammatory factors were measured in plasma samples preoperatively and at 0.5 postoperative hour. IL-6 levels were starkly reduced in the BCAA group compared with control animals postoperatively at 0.5 h (*P* < 0.01), and elevated in both the BCAA and Con groups postoperatively compared with preoperative values (Fig. 4).

### Regulatory effects of BCAA on glucose metabolism genes in the liver

**Fig. 5:**
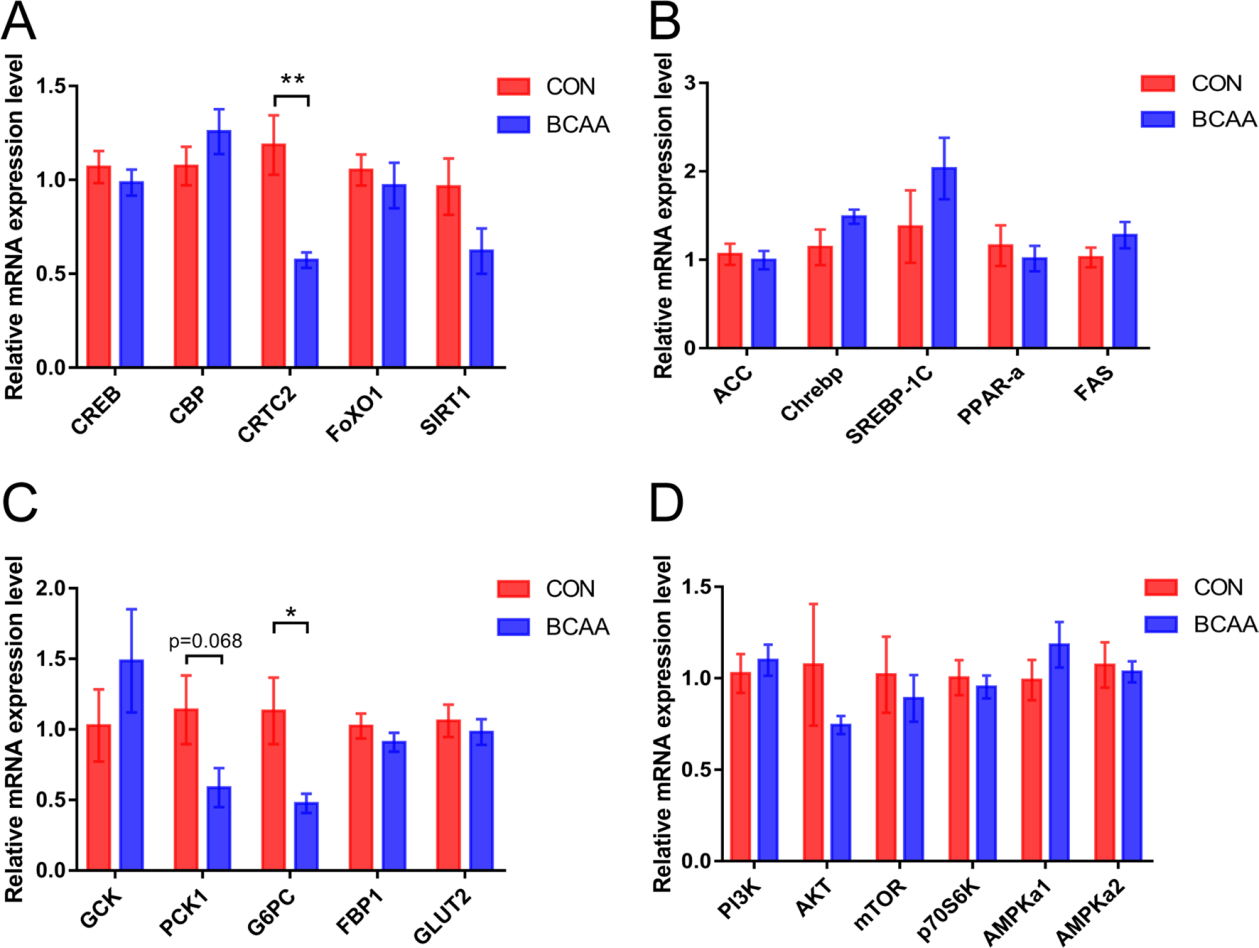
Effects of BCAA administration on the expression of mRNA related to glucose metabolism in the liver. **A** qPCR analysis of gluconeogenesis genes related mRNA expression (n = 8). **B** qPCR analysis of glucose and lipid metabolism genes related mRNA expression (n = 8). **C** qPCR analysis of glucose metabolism genes related mRNA expression (n = 8). **D** qPCR analysis of insulin signaling pathway related genes mRNA expression (n = 8). Data are shown as mean ± SEM. *P < 0.05, **P < 0.01. BCAA: branched-chain amino acids, CON: control, CREB: cAMP response element binding protein, CBP: CREB-binding protein, CRTC2: CREB regulated transcriptional coactivator 2, FoxO1: Forkhead box O1, SIRT1: Sirtuin 1, ACC: Acetyl-CoA carboxylase, Chrebp: Carbohydrate responsive element binding protein, SREBP-1C: Sterol response element binding protein 1C, PPAR-a: Peroxisome proliferator-activated receptor-a, FAS: fatty acid synthase, GCK: glucokinase, PCK1: phosphoenolpyruvate-carboxykinase 1, G6PC: glucose-6phosphatase, FBP1: fructose-1,6-bisphosphatase, GLUT2: glucose transporter 2, PI3K: phosphatidylinositol 3 kinase, AKT: Protein kinase B, mTOR: mammalian target of rapamycin, P70S6K: P70 ribosomal protein S6 kinase, AMPKa1: AMP-activated protein kinase a1, AMPKa2: AMP-activated protein kinase a2

qPCR assays showed that the BCAA group had decreased CRTC2 mRNA levels in the liver tissue postoperatively in comparison with control animals (*P* < 0.01, Fig. 5A), and also decreased the transcription of G6PC, one of the key enzymes of hepatic gluconeogenesis (*P* < 0.05, Fig. 5C), Another key enzyme of hepatic gluconeogenesis, PCK1, was also downregulated by BCAA, but non-significantly.

### Regulatory effects of BCAA on glucose metabolism proteins in the liver

**Fig. 6:**
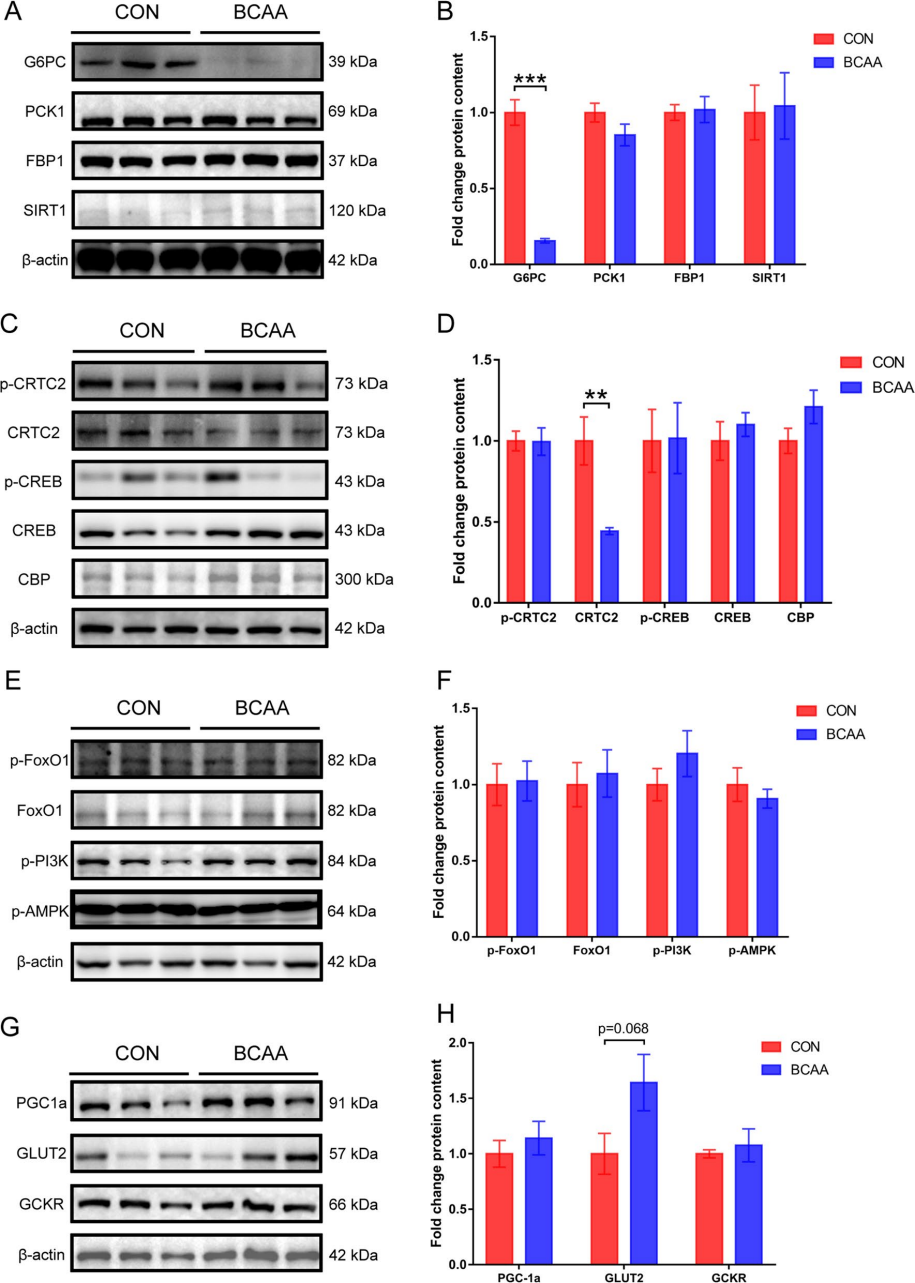
Western Blot of selected liver proteins related to glucose metabolism. **A** Western blot analyses of G6PC, PCK1, FBP1, SIRT1 and β-actin proteins (n = 6). **B** G6PC, PCK1, FBP1 and SIRT1 protein levels were quantified by densitometric analysis using ImageJ software (n = 6). **C** Western blot analyses of p-CRTC2, CRTC2, p-CREB, CREB, CBP and β-actin proteins (n = 6). **D** p-CRTC2, CRTC2, p-CREB, CREB and CBP protein levels were quantified by densitometric analysis using ImageJ software (n = 6). **E** Western blot analyses of p-FoxO1, FoxO1, p-PI3K, p-AMPK and β-actin proteins (n = 6). **F** p-FoxO1, FoxO1, p-PI3K and p-AMPK protein levels were quantified by densitometric analysis using ImageJ software (n = 6). **G** Western blot analyses of PGC-1a, GLUT2, GCKR and β-actin proteins (n = 6). **H** PGC-1a, GLUT2 and GCKR protein levels were quantified by densitometric analysis using ImageJ software (n = 6). Data are shown as mean ± SEM. **P < 0.01, ***P < 0.001. BCAA: branched-chain amino acids, CON: control, G6PC: glucose-6phosphatase, PCK1: phosphoenolpyruvate-carboxykinase 1, FBP1: fructose-1,6-bisphosphatase, SIRT1: Sirtuin 1, CRTC2: CREB regulated transcriptional coactivator 2, CREB: cAMP response element binding protein, CBP: CREB-binding protein, FoxO1: Forkhead box O1, PI3K: phosphatidylinositol 3 kinase, AMPK: AMP-activated protein kinase, PGC1a: Peroxisome proliferator-activated receptor gamma coactivator-1 alpha, GLUT2: glucose transporter 2, GCKR: glucokinase regulatory protein

Western blot assessment of glucose metabolism-related proteins in the liver tissue postoperatively showed that the BCAA group had decreased protein expression levels of G6PC, a key enzyme of gluconeogenesis, in comparison with control animals, with statistical significance of gray-scale values (*P* < 0.001) (Fig. 6A, B). The protein amounts of gluconeogenic regulation-related CRTC2 in postoperative liver tissues were lower in the BCAA group in comparison with control animals, with statistical significance in gray-scale analysis (*P* < 0.01, Fig. 6C, D). There was a tendency of elevation in the BCAA group compared with control animals in the hepatic glucose transport-related protein GLUT2 (Fig. 6G, H), but the values were not statistically different.

## Discussion

**Fig. 7:**
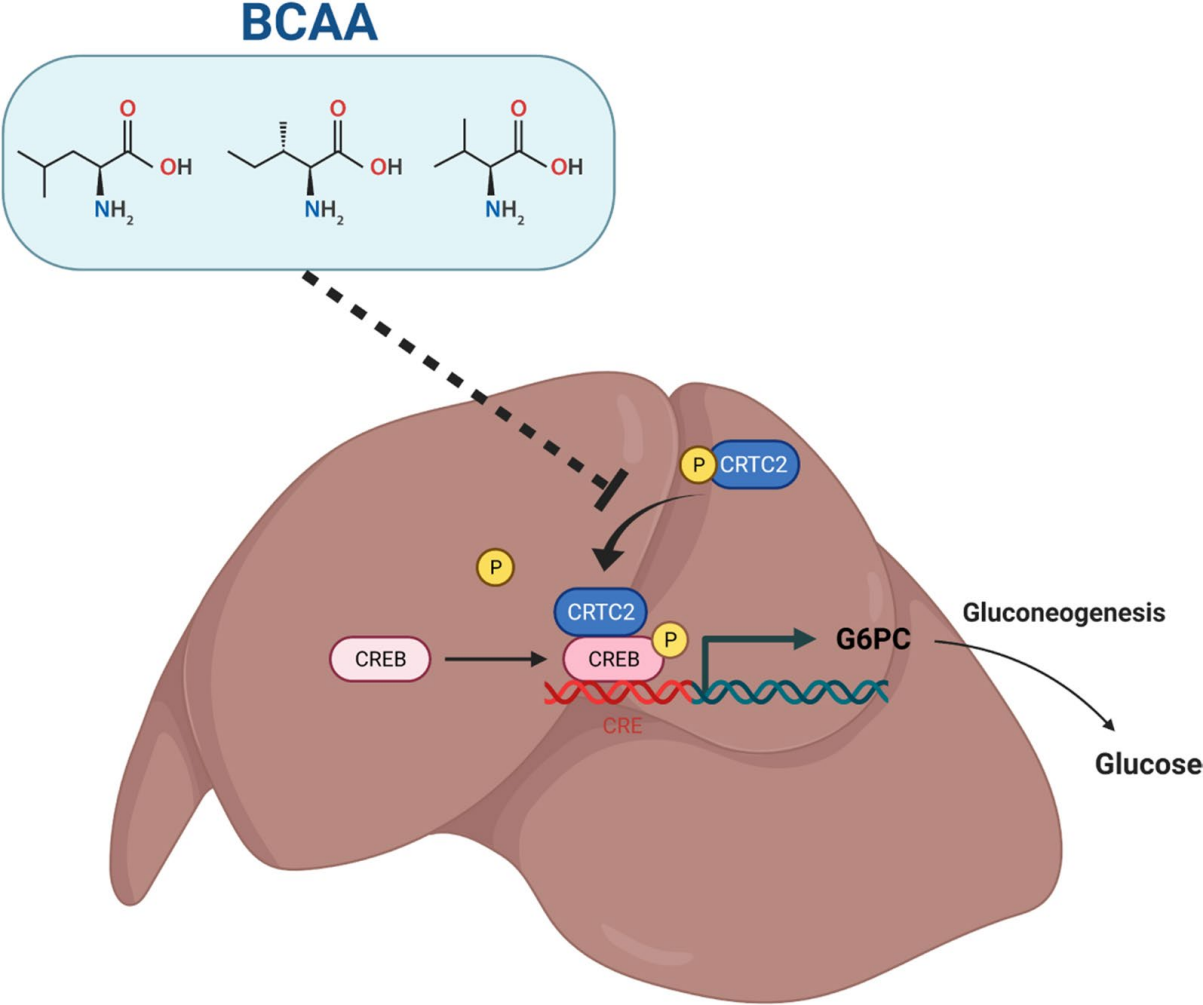
Schematic diagram of the effect of BCAA on alleviating postoperative liver gluconeogenesis. BCAA inhibits dephosphorylation of CRTC2, thereby reducing CRTC2 binding to p-CREB, decreasing transcription and translation of G6PC, decreasing hepatic gluconeogenesis and reducing postoperative insulin resistance. BCAA: branched-chain amino acids, CREB: cAMP response element binding protein, CRTC2: CREB regulated transcriptional coactivator 2, G6PC: glucose-6phosphatase

This study investigated the effect of oral BCAA on postsurgical insulin resistance in rats. We proposed for the first time the hypothesis that 2-h preoperative BCAA intake reduces postoperative insulin resistance and postoperative hyperglycemia by decreasing hepatic gluconeogenesis. A schematic diagram depicting the effect of BCAA in alleviating postoperative liver gluconeogenesis is provided (Fig. 7). BCAA inhibited CRTC2 dephosphorylation and reduced CRTC2 binding to phosphorylated CREB, which in turn reduced the expression of G6PC, a key enzyme of gluconeogenesis, resulting in reduced gluconeogenesis.

The key finding of this study was that BCAA intake improved postoperative insulin resistance and reduced postoperative hyperglycemia, by decreasing hepatic gluconeogenesis. To acquire insights into the possible molecular mechanisms of improved insulin sensitivity, qPCR and Western blot were performed to assess liver tissue specimens. Specifically, we found that BCAA downregulated hepatic CRTC2 and G6PC mRNAs, which in turn reduced the rate of hepatic gluconeogenesis. It was previously reported that leucine promotes insulin secretion [12], which may be responsible for postoperative glucose reduction. However, postoperative insulin levels were markedly different between the BCAA and CON groups. Similarly, insulin signaling pathways were not significantly altered in liver, muscle and fat tissue specimens after surgery. Glucagon, another key factor regulating blood glucose levels [45], was also examined for its preoperative and postoperative levels. We found that postoperative glucagon levels were markedly elevated in both groups in comparison with preoperative levels, but there was no marked difference between the two groups, suggesting that glucagon is not responsible for the difference in postoperative blood glucose amounts. The experimental results of GTT and ITT showed that postoperative glucose tolerance and insulin sensitivity are significantly improved by BCAA gavage. Interestingly, the PTT test revealed gluconeogenesis was starkly reduced in the BCAA gavage group compared with control animals. IL-6 has been shown in many studies to have an important relationship with the development of postoperative insulin [41, 46]. In this study, oral BCAA treatment reduced IL-6 levels in postoperative plasma samples, which may be responsible for the reduction of postoperative gluconeogenesis. Few studies have reported that BCAA reduces IL-6 levels, and further investigations are needed to examine the underpinning mechanism.

The liver is critical in maintaining glucose homeostasis for its capability of storing and producing endogenous glucose [47]. Hepatic glucose regulation is controlled by hormones and nutrition. The process of hepatic glucose production includes gluconeogenesis, glycogenolysis and glycolysis. In the fasting state, glucose production is mainly the result of gluconeogenesis. CRTC2 represents a coactivator that is specific to CREB and has an important regulatory function in controlling hepatic gluconeogenesis and maintaining the stability of fasting blood glucose [37]. Dephosphorylation of CRTC2 Ser171 activates and promotes its binding to CREB, which binds to G6PC and PCK1, key downstream enzymes regulating the rate of gluconeogenesis, to promote hepatic gluconeogenesis [38, 39]. In the present study, BCAA inhibited hepatic gluconeogenesis by reducing CRTC2 and G6PC gene and protein expression levels, resulting in a significant reduction in blood glucose, which has never been reported previously. In this paper, the cause of the down-regulation of G6PC is thought to be caused by BCAA reducing the expression of CRTC2. CRTC2 is a co-activator of CREB, and as the expression level of CRTC2 decreases, so does the transcriptional activity of CREB, which leads to a decrease in the expression of its downstream target gene G6PC. In the basal state, phosphorylated CRTC2 binds to the 14-3-3 protein and is isolated in the cytoplasm [48]. Inhibition of Salt-Inducible Kinase 2 (SIK2, which phosphorylate CRTC2) activity in response to calcium ion and cAMP stimulation promoted dephosphorylation of CRTC2, leading to the entry of CRTC2 from the cytoplasm into the nucleus and activation of its activity in combination with phosphorylated CREB. BCAA may reduce CRTC2 expression levels by increasing CRTC2 phosphorylation or by inhibiting CRTC2 dephosphorylation. Given that no difference in phosphorylated CRTC2 was detected in this manuscript, we speculate that it is possible that BCAA inhibits the dephosphorylation of CRTC2 (indicated by the dashed line in Fig. 7). Whether BCAA affects SIK2 activity has not been reported, and if BCAA can activate SIK2, it can also increase the phosphorylation of CRTC2, making the expression of non-phosphorylated CRTC2 level decreased. However, in the present manuscript, no difference in phosphorylated CRTC2 was detected, and the speculation is not valid. In addition, acetylation can also affect the expression level of CRTC2. Phosphorylation of P300 Ser89 can deacetylate and then inactivate CRTC2. Our present manuscript provides a possible hypothesis and the mechanism of CRTC2 reduction by BCAA needs to be further investigated. There is another important possible mechanism, which is mTOR, and according to the literature, BCAA can activate mTOR, while CRTC2 can be phosphorylated by mTOR at Ser136 [48]. Since the molecular weight of mTOR is too large, direct detection by Western Blot is difficult and is often achieved by detecting PI3K, AKT, and P70S6K in the classical upstream and downstream pathways of mTOR [49]. In the present manuscript, no differences in key proteins of the upstream and downstream pathways of mTOR were detected, suggesting to us that BCAA activation of mTOR, which in turn promotes phosphorylation of CRTC2, may not be responsible for the reduction of active CRTC2. In this work, BCAA’s effect on glucose metabolism was assessed by the pyruvate tolerance test, which reflects changes in glucose production from pyruvate, a key gluconeogenesis precursor. This study has several limitations. It is unclear which of leucine, isoleucine or valine decreases gluconeogenesis, or if all three are jointly involved. We did not perform some reverse experiments to elucidate the main mechanism by which BCAA reduces hepatic gluconeogenesis. It would be fantastic to inject IL-6 in the BCAA group to reverse the results of Fig. 2, or to inject IL-6 receptor blockers in the control group to achieve close postoperative glucose levels in both groups. Or, again, whether the mechanism can be demonstrated in reverse by applying an activator of the hepatic gluconeogenic pathway.

Further research is needed to clarify the mechanism of BCAA-related reduction of PIR, which may involve an insulin-independent pathway. The present findings add to the current wealth of knowledge that BCAA improves postoperative glucose metabolism. It may be potentially important to elucidate the regulatory role of BCAA in liver gluconeogenesis, which may be a future research topic.

## Conclusions

In conclusion, BCAA administration represses gluconeogenesis in the liver by reducing CRTC2 and G6PC gene and protein expression levels, thereby leading to a hypoglycemic effect in rats. Our study revealed that BCAA lowers blood glucose levels by reducing liver gluconeogenesis without significant elevation of plasma insulin levels. We can anticipate that preoperative BCAA supplementation may be a means for preventing postoperative insulin resistance.

## Supplementary Information

**Additional file 1. **Postoperative blood glucose in the surgical or sham surgical group treated with BCAA or not.

## Data Availability

The datasets used or analysed during the current study are available from the corresponding author on reasonable request.

## Abbreviations

PIR: Postoperative insulin resistance
BCAA: Branched-chain amino acids
CRTC2: CAMP-response element-binding protein-regulated transcription coactivator 2
G6PC: Glucose-6-phosphatase
AIR: Acute insulin resistance
POC: Preoperative oral carbohydrate
ERAS: Enhanced Recovery After Surgery
CREB: CAMP-response element-binding protein
PCK1: Phosphoenolpyruvate carboxykinase 1
CON: Control
HOMA: Homeostasis model assessment
GTT: Glucose tolerance test
ITT: Insulin tolerance test
PTT: Pyruvate tolerance test
AUC: Area under the curve
TNF-α: Tumor necrosis factor-α
IFN-γ: Interferon γ
GM-CSF: Granulocyte–macrophage colony-stimulating factor
SEM: Standard error of the mean
GLUT2: Glucose transporter 2
AKT: Protein kinase B
P70S6K: P70 ribosomal protein S6 kinase
CBP: CREB-binding protein
FoxO1: Forkhead box O1
SIRT1: Sirtuin 1
ACC: Acetyl-CoA carboxylase
Chrebp: Carbohydrate responsive element binding protein
SREBP-1C: Sterol response element binding protein 1C
PPAR-a: Peroxisome proliferator-activated receptor-a
FAS: Fatty acid synthase
GCK: Glucokinase
FBP1: Fructose-1,6-bisphosphatase
PI3K: Phosphatidylinositol 3 kinase
mTOR: Mammalian target of rapamycin
AMPK: AMP-activated protein kinase
PGC1a: Peroxisome proliferator-activated receptor gamma coactivator-1 alpha
GCKR: Glucokinase regulatory protein
SIK2: Salt-Inducible Kinase 2
