# Supplementary material for: Preoperative administration of branched-chain amino acids reduces postoperative insulin resistance in rats by reducing liver gluconeogenesis
Source: Nutr Metab (Lond). 2022 Nov 29;19:78. doi: 10.1186/s12986-022-00710-3 (PMC9706859; doi:10.1186/s12986-022-00710-3)
Supplement: Supplementary file 1 — Additional file 1. Postoperative blood glucose in the surgical or sham surgical group treated with BCAA or not. [file 12986_2022_710_MOESM1_ESM.docx]

**Postoperative blood glucose in the surgical or sham surgical group treated with BCAA or not.**


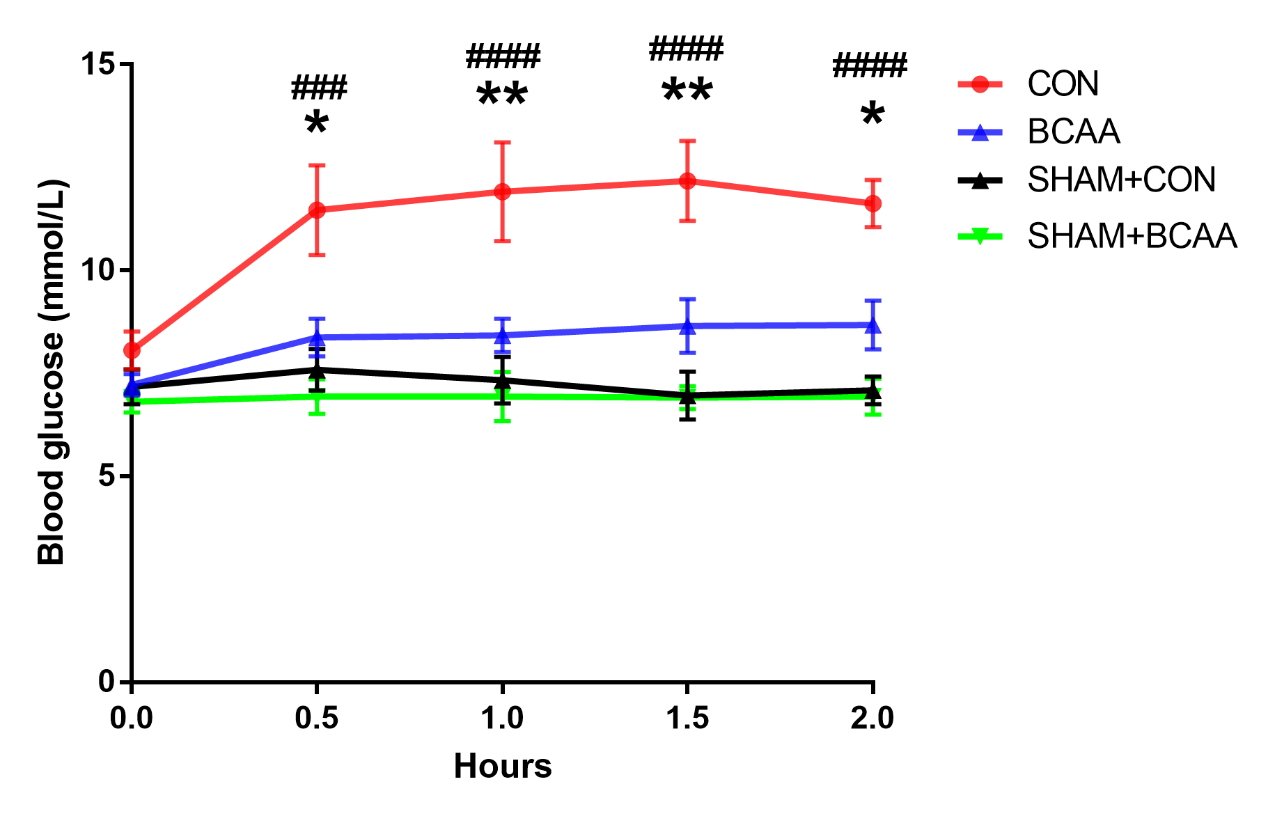


Postoperative blood glucose. Data are shown as mean ± SEM (n=8). BCAA: branched-chain amino acids, CON: control, SHAME: sham-operated group. Data were analyzed by two-way analysis of variance, with the Bonferroni correction for multiple comparisons. P<0.05 was considered to be statistically significant. * P < 0.05 and **P < 0.01 BCAA versus CON group; ^###^P < 0.001 and ^####^P < 0.0001 CON versus SHAM+CON group.
